# Development and evaluation of a tool for the assessment of footwear characteristics

**DOI:** 10.1186/1757-1146-2-10

**Published:** 2009-04-23

**Authors:** Christian J Barton, Daniel Bonanno, Hylton B Menz

**Affiliations:** 1 School of Physiotherapy, Faculty of Health Sciences, La Trobe University, Bundoora, Victoria, Australia; 2 Musculoskeletal Research Centre, Faculty of Health Sciences, La Trobe University, Bundoora, Victoria, Australia; 3 Department of Podiatry, Faculty of Health Sciences, La Trobe University, Bundoora, Victoria, Australia

## Abstract

**Background:**

Footwear characteristics have been linked to falls in older adults and children, and the development of many musculoskeletal conditions. Due to the relationship between footwear and pathology, health professionals have a responsibility to consider footwear characteristics in the etiology and treatment of various patient presentations. In order for health professionals and researchers to accurately and efficiently critique an individual's footwear, a valid and reliable footwear assessment tool is required. The aim of this study was to develop a simple, efficient, and reliable footwear assessment tool potentially suitable for use in a range of patient populations.

**Methods:**

Consideration of previously published tools, other footwear related literature, and clinical considerations of three therapists were used to assist in the development of the tool. The tool was developed to cover fit, general features, general structure, motion control properties, cushioning, and wear patterns. A total of 15 participants (who provided two pairs of shoes each) were recruited, and assessment using the scale was completed on two separate occasions (separated by 1 – 3 weeks) by a physiotherapist and a podiatrist on each participant's dominant foot. Intra-rater and inter-rater reliability were evaluated using intra-class correlation coefficients (ICCs) (model 2, 1) and the 95% limits of agreement (95% LOAs) for continuous items, and percentage agreement and kappa (κ) statistics for categorical items.

**Results:**

All categorical items demonstrated high percentage agreement statistic for intra-rater (83 – 100%) and inter-rater (83 – 100%) comparisons. With the exception of last shape and objective measures used to categorise the adequacy of length, excellent intra-rater (ICC = 0.91 – 1.00) and inter-rater reliability (ICC = 0.90 – 1.00) was indicated for continuous items in the tool, including the motion control properties scale (0.91 – 0.95).

**Conclusion:**

A comprehensive footwear assessment tool with good face validity has been developed to assist future research and clinical footwear assessment. Generally good reliability amongst all items indicates that the tool can be used with confidence in research and clinical settings. Further research is now required to determine the clinical validity of each item in various patient populations.

## Background

Footwear has been used by humans for thousands of years. Footwear characteristics have been developed and modified to provide protection from the environment, conform with fashion, assist function, accommodate foot deformities, and treat musculoskeletal injury [1]. Various footwear characteristics have been linked to falls in older people [2-7] and in children [8], and the development of multiple conditions including osteoarthritis of the foot [9,10] and knee [11], low back pain [12,13], foot ulcerations and amputations [14], and foot deformities such has hallux valgus and hammer toes [15].

Due to the relationship between footwear and pathology, health professionals have a responsibility to consider footwear characteristics in the aetiology and treatment of various patient presentations. Many issues related to foot, lower limb, and low back conditions can often be addressed by changing or modifying footwear, with or without the use of foot orthoses. When considering foot orthoses prescription, the Australian Podiatry Council's clinical guidelines [16] state that the influence of footwear style and fit on the patient's clinical condition should be addressed first. If choosing to implement foot orthoses, the suitability of a patient's footwear to accommodate the orthoses must first be assessed [16]. In some cases, changing a patient's footwear may be the only intervention required [17].

In order for health professionals to accurately and efficiently critique an individual's footwear and provide advice, a valid and reliable footwear assessment tool is required. The availability of such tools within the literature is currently limited. The *Footwear Checklist *[17] was recently published to provide guidance to health professionals when assessing patients' footwear. The *Footwear Suitability Scal*e [18], and the nine item *Footwear Assessment Score *[19] have also been published in the literature to assess suitability of footwear for diabetic patients and children respectively. Unfortunately, none of these three assessment tools were published with accompanying reliability evaluation for the items contained within them. Menz and Sherrington [20] developed the seven item *Footwear Assessment Form *as a simple clinical tool to assess footwear characteristics related to postural stability and falls risk factors in older adults. The scale was reported to possess generally high reliability for intra-rater and inter-rater comparisons. However, like other published footwear assessment tools [18,19], it is intended for a specific population, limiting its broader application. The purpose of this investigation was to develop a simple, efficient, and reliable footwear assessment tool to assess a broad range of footwear characteristics and which is potentially suitable for use in a range of patient populations.

## Methods

### Development of the footwear assessment tool

Consideration of previously published tools [17-20], other footwear-related literature, and clinical experience of the participating researchers were used to assist in the development of the tool (see Additional material file 1). The three researchers included a physiotherapist with three years clinical experience, a podiatrist with nine years clinical experience and a podiatrist with 15 years clinical and research experience. Footwear characteristics considered to be important in the development and treatment of varying foot, lower limb, and low back conditions, as well as falls and diabetes-related issues were included in the tool. To allow a more objective measure of footwear quality in regard to motion control prior to consideration of foot orthoses prescription, a motion control properties scale was also devised using items extracted from the tool. An explanation of each of the six items and the justification for inclusion is now outlined and measurement techniques described. Photographs of the measurement techniques related to each item in the scale can be found in Additional material file 1.

### Item 1. Fit

Poorly fitting shoes have been linked to falls [4,15,21,22], foot pain [23,24], pressure lesions in patients with diabetes [23,25,26], neuromas [15], corns and calluses [27] and toe deformity in older people [24]. All measures of fit were taken in weight-bearing (WB) due to the splaying and elongation of the foot which occurs when moving from a non-weight-bearing (NWB) to WB position [28,29]. Three aspects of fit were included in the scale including length, width and depth.

#### Length – rule of thumb

A gap of between 10 and 20 mm [14,17-19], or a thumb's width [1,27] from the longest toe to the front of the shoe are common recommendations in the literature. For this item, therapist palpation was used to categorise footwear as too short (< 1/2 a thumb's width), good (between a 1/2 and 1 1/2 of a thumb's width), or too long (> 1 1/2 of a thumb's width). A second, more objective, length measurement was also included in the tool. This involved measuring the length inside the shoe using a flexible plastic straw, measuring foot length using a custom built Brannock-style device, and calculating the difference. The difference was then compared to the footwear owner's thumb width (measured by ruler at the base of the nail) and categorised in the same way as the palpation method. This measurement was taken second so it would not bias the more subjective palpation method. This method allows a more quantifiable measure of length adequacy, and allows for length assessment of footwear which does not allow palpation through the toe box (e.g. steel capped boots).

#### Width – grasp test

To measure the adequacy of footwear width, grasping of the upper over the metatarsal heads was used to categorise footwear as too wide (excessive bunching of the upper), good (slight bunching of the upper), or too narrow (tight, taught upper unable to be grasped) [1,14].

#### Depth

Consideration of the ability of the toes and joints to move freely, and the absence of pressure on the dorsal aspect of the toes and nails was considered to categorise depth as adequate or too shallow [17].

### Item 2. General features

#### Age of shoe

The age of the shoe is important to evaluate the significance of wear patterns, and to determine when replacement may be required. The therapist may consider this information in relation to other subjective examination information such as occupation or intended purpose of the footwear, and also frequency of wear. This was based on participants' self-report of the age of the shoe.

#### Footwear type

Footwear type has been linked to diminished balance and falls in older people [2,4-6]. This item was taken from the *Footwear Assessment Form *[20]. A sheet containing representative diagrams of each category was used to improve reliability and assist decisions on this item (see Additional material file 1).

#### Materials (upper)

The upper is most commonly constructed from leather, but can also be made from various synthetic materials [14]. Leather is more expensive than synthetic materials but is considered superior due to its durability, greater breathability and subsequent ability to prevent fungal growth, and ability to mould to deformities of the foot without resulting in pressure areas and ulcer formation in patients with diabetes [14,17,30]. Synthetic materials can be made to be more breathable (e.g. mesh), however, at the expense of durability. Therefore, materials (upper) were categorised as leather, synthetic, mesh, or other.

#### Materials (outsole)

Rubber, plastic, and leather can all be used in construction of footwear outsoles. Rubber outsoles are thought to be superior due their ability to increase slip resistance, thereby reducing the risk of falls in older people [4,31] and in children [8]. However, in some instances, leather or plastic may be used to improve the aesthetics of footwear. This item was categorised as rubber, plastic, leather, or other.

#### Weight/length ratio

The ratio of weight/length was considered by the three participating researchers to be important in influencing gait efficiency. The weight of footwear was measured in grams using Homemaker™ digital scales (+/- 1 gram). Length of the shoe was measured in millimeters from the most posterior aspect of the upper heel cup to the most distal aspect of the upper toe box using a custom made Brannock-style device. The ratio was determined by dividing the weight by the length. No categories were devised for this item as there is currently no evidence or previous tools upon which to base them.

### Item 3. General structure

#### Heel height

Wearing high-heeled shoes has been reported to diminish static and dynamic balance [32-36], and increase the risk of falling in older people [7]. High-heeled shoes have also been implicated in the development of low back pain [13], osteoarthritis of the knee [11,37] and forefoot [9,10], and hallux valgus and calluses in older people [27]. Categories for increased heel height were taken from the *Footwear Assessment Form *(0 to 2.5 cm, 2.6 to 5.0 cm, or > 5.0 cm) [20] and match previous recommendations for older people [22]. Measurement was recorded as the average of the height medially and laterally from the base of the heel to the centre of the heel-sole interface.

#### Forefoot height (measured at point of first and fifth metatarsophalangeal joints)

Since the relationship between heel height, forefoot height, and footwear length and not heel height alone will effect the position of the foot in the shoe, forefoot height was also measured. This measurement was taken at the level of both the first and fifth metatarsophalangeal joints and the average of both recorded. The measurement was then categorised as 0 to 0.9 cm, 1.0 to 2.0 cm or > 2.0 cm.

#### Normalised longitudinal profile (heel – forefoot difference, or pitch)

The longitudinal profile was recorded as the difference between heel height and forefoot height (also referred to as pitch). This item was categorised as flat (0 to 0.9 cm), small heel rise (1.0 to 3.0 cm), or large heel rise (> 3.0 cm). This measure was then normalised by dividing it by the length of the shoe. This normalised measure takes into account all factors which will determine the position of the foot in the shoe. For example, a 3.0 cm heel height will plantarflex the foot more in a shoe containing a 1.5 cm forefoot height when compared to a shoe containing a 2.5 cm forefoot height. Likewise, variations in heel:forefoot profile will plantarflex a shorter foot more than a longer foot.

#### Last shape

The last of a shoe is considered important to accommodate variations in foot type. Whilst a straight last is thought to accommodate a pronated foot type and assist motion control, a curve last is thought to better accommodate a more supinated foot and optimise gait efficiency [1]. The last shape was measured by bisecting the heel and forefoot areas on the shoe sole, and then measuring the angular difference between the two using a plastic goniometer with its axis positioned in the centre of the shoe. The three categories devised were straight (0 to 5°), semi-curved (5 to 15°), and curved (> 15°). The angular values for each category were devised by consideration of measurements from a wide range of shoes. A visual observation to categorise the last shape was made prior to using the goniometer.

#### Fixation of upper to sole

Common methods for fixing the upper of a shoe to the sole include board lasting and slip (stitch) lasting. Board lasting involves using a board, usually made out of light weight wood, which is glued to both the upper and the sole in order to combine them, whilst slip lasting involves stitching the upper directly to the sole. Board lasting footwear is thought to provide greater stability, however, it is heavier, may be less comfortable and is considered a more expensive manufacturing process than slip lasting [1]. The two methods can also be combined (combination last) to provide stability to the rearfoot whilst optimising weight, comfort and flexibility in the forefoot [1]. This item was categorised as board lasted, slip lasted, or combination lasted.

#### Forefoot sole flexion point

A flexion point distal to the level of the first metatarsophalangeal joint (1^st ^MPJ) may limit gait efficiency due to altered kinematics which result from inhibition of normal 1^st ^MPJ function [38]. A flexion point proximal may jeopardise the shoe's stability. To measure this, a sagittal bending force was applied to the shoe's sole and the point at which the bend occurred was noted. This item was categorised as: at level of MPJs, proximal to MPJs, or distal to MPJs.

### Item 4. Motion control properties

Motion control properties of footwear are considered important in falls prevention [19,38-40], treatment of patients with diabetes [26] and rheumatoid arthritis [40], and treatment of musculoskeletal injuries [1,39,41,42]. A range of footwear properties may assist motion control of the foot. These include fixation of the upper to the foot, heel counter stiffness, and midfoot rigidity [1]. More recently, in athletic footwear, midsoles made of multiple densities (with the highest density located medially) have been developed in an attempt to further improve motion control of the shoe [1,15,39,41].

#### Multiple density sole

This item was categorised as single density or multiple density.

#### Fixation

Laces are considered the most optimal form of fixation as they allow the fit of the shoe to be individually adjusted [1,15], however, they can be difficult for some patients to manage. Other alternatives in these cases include straps/buckles, Velcro™, and zips. This item was taken from the *Footwear Assessment Form *[20], and categorised as none, laces, straps/buckles, Velcro™, or zips.

#### Heel counter stiffness

Heel counter stiffness is an important consideration when rearfoot motion control is desired [15]. A stiff heel counter is also thought to improve balance [4,14,20]. This item was taken from the *Footwear Assessment Form *[20]. Categories included none, minimal (> 45°), moderate (< 45°), or rigid (< 10°). To measure this, the heel counter was pressed with firm force approximately 20 mm from its base and the angular displacement estimated.

#### Midfoot sole sagittal stability

Since the midfoot is required to form a rigid lever during propulsion, footwear stability in this area was thought to be an optimal motion control property. This item was taken from the *Footwear Assessment Form *(referred to as 'longitudinal sole rigidity') [20], with the categories minimal (> 45°), moderate (< 45°), or rigid (< 10°). To measure this, both the rearfoot and forefoot components of the shoe were grasped and attempts were made to bend the shoe at the midfoot in the sagittal plane.

#### Midfoot sole frontal stability (torsion)

Torsional stability at the midfoot was also considered important to determine the level of midfoot motion control provided by the shoe. This item was given the same categories as the midfoot sole sagittal stability item. To measure this, both the rearfoot and forefoot components of the shoe were grasped and attempts were made to twist the shoe at the midfoot in the frontal plane.

#### Scale for motion control properties

To develop a continuous scale to assess the quality of footwear in relation to motion control properties, each category from the motion control properties items were assigned a score. The score allocations for all categories from each item are outlined in Additional material file 1, with the possible total score ranging from 0 to 11, and greater scores indicating superior motion control properties. Therefore, footwear which scores 11 would be considered to possess optimal motion control properties, whilst footwear which scores 0 would be considered to possess least optimal motion control properties.

### Item 5. Cushioning

Greater shock absorbing properties (enhanced cushioning) in footwear have been considered important in overuse injury prevention [41-44]. Although increased cushioning is thought to improve shock absorption characteristics of footwear and decrease injury rates, current evidence to support this association is not strong [45-47]. Previous reports on the effect of footwear midsole density (often modified to enhance cushioning and optimise motion control) on balance have varied, although impaired beam walking ability [48] reduced medio-lateral stability [34] and reduced step length [36] with softer midsoles in older people has been reported. Characteristics which may alter shock absorption properties of footwear are thought to include the presence of cushioning systems, midsole hardness, and heel sole (interface of heel to sole of shoe) hardness.

#### Presence of cushioning system

Many modern footwear designs include the addition of specifically designed cushioning systems most commonly made from air or gel pockets. This item was categorised as none, heel, or heel/forefoot.

#### Lateral midsole hardness

The lateral aspect of the heel is generally the first part of the foot to strike the ground during normal walking gait, making the properties of the shoe at this aspect theoretically important to initial shock attenuation. This item was subjectively categorised as soft, firm, or hard. Under firm pressure from the examiner's thumb, minimal to no indentation (< 0.5 mm) was scored hard, moderate indentation (0.5 – 1.5 mm) was scored firm, and marked indentation (> 1.5 mm) was scored soft. Due to previously reported poor reliability for a similar item [20], recommendations to obtain Shore A durometer hardness measurements with a penetrometer (Yuequing Handpi Instruments Co., Ltd) were also followed, providing a quantitative measure of lateral midsole hardness. These measurements were taken second so they did not bias subjective measurements. Since only small translational differences in placement of the penetrometer produced different durometer measurements, this measure was recorded as the average of three separate readings.

#### Medial midsole hardness

In footwear with multiple density midsoles the density of the medial midsole was scored using the same subjective categories, and objective Shore A durometer measurements used in lateral midsole hardness item.

#### Heel sole hardness

The same subjective categories and objective Shore A durometer measurements used in lateral and medial midsole hardness items were used for this item. This measurement was taken at the foot (inferior heel)-shoe interface.

### Item 6. Wear patterns

Wear patterns of footwear can provide health professionals with some insight into how an individual's foot is functioning in the shoe [17,49], and provide guidance as to when a shoe has become unsafe or requires replacement. Wear pattern items included were upper, midsole, tread pattern, and outsole.

#### Upper

This item was categorised as neutral, medial tilt greater than 10°, which may indicate excessive pronation, or lateral tilt greater than 10°, which may indicate excessive supination [49].

#### Midsole

This item was categorised as neutral, medial tilt (medial midsole compression), which may indicate excessive pronation, or lateral tilt (lateral midsole compression), which may indicate excessive supination [48].

#### Tread pattern

Since textured tread pattern has been considered an important falls prevention characteristic [4,14,20,31], the presence and wearing of the outersole was included in the scale. Tread pattern was divided into two items consisting of textured or smooth; and no wear, partly worn, or fully worn.

#### Outersole wear pattern

This item was categorised as none, normal (i.e. starting posterior lateral heel and moving medially towards the first ray distally along the shoe), medial (greater medial than lateral wear at the heel and forefoot), which may indicate excessive pronation, or lateral (greater lateral than medial wear at the heel and forefoot), which may indicate excessive supination [49].

### Data collection procedure

Ethical approval was granted by La Trobe University's Faculty of Health Sciences Human Ethics Committee. Information sheets were provided and written consent obtained from each participant prior to the commencement of the study. A total of 15 staff members from the Faculty of Health Sciences, La Trobe University were recruited to the study. Each participant was required to contribute two pairs of their own footwear for assessment (i.e. number of different footwear totaled 30). Guidance on what type of footwear to contribute was provided by the investigators to ensure the scale was tested on a wide range of footwear. Footwear were assessed on the participant's dominant foot only, and assessment was carried out by a physiotherapist (rater 1), and a podiatrist (rater 2). The same footwear was then retested between one and three weeks later. During application of the tool, both raters were blinded to each other's results, and their own previous results.

### Statistical analysis

Since only three shoes contained multiple density midsoles, lateral and medial midsole hardness items were combined for data analysis. Intra-rater and inter-rater reliability for all continuous data were evaluated using intra-class correlation coefficients (ICCs) (model 2,1). Intra-class correlation coefficients above 0.90 were considered excellent, 0.75 to 0.90 considered good, and below 0.75 considered poor to moderate [50]. The 95% limits of agreement (95% LOAs) was calculated for continuous measures so that potential errors for each item could be quantified in units of its measurement [50]. So that measurement errors could be considered in context, the range of each measure across included footwear was also reported. Intra-rater and inter-rater reliability for all categorical data was evaluated using percentage agreement, and kappa (κ) statistics [51,52]. Kappa values above 0.80 were considered excellent, 0.60 to 0.80 considered substantial, 0.40 to 0.60 considered moderate, and below 0.40 considered poor to fair [52].

## Results

Footwear types contributed by participants included walking shoes, athletic shoes, oxford shoes, moccasins, boots, high heels, thongs (flip-flops), slippers, court shoes, and sandals. The range for each quantitative measure from the included footwear can be found in Table 1.

**Table 1:** Range for each continuous measurement.

Variable	Range
*Fit*	
Foot length	273 – 274 mm
Inside shoe length (straw)	221 – 289 mm
Inner shoe length – foot length	-8 – 22 mm
Thumb width	17 – 24 mm
*General*	
Weight	86 – 591 g
Length	231 – 301 mm
Weight/length	0.36 – 2.05
*General structure*	
Heel height	4 – 82 mm
Forefoot height	2 – 26 mm
Longitudinal profile	0 – 79 mm
Normalised longitudinal profile	0.000 – 0.322
Last shape	0 – 14°
*Motion control properties*	
Number of laces	3 – 7
Motion control sub-scale	0 – 11
*Cushioning system*	
Midsole durometer	34 – 100
Heel sole durometer	10 – 87

### Intra-rater reliability

Intra-rater ICCs and 95% LOAs for quantitative measures are shown in Table 2. Similar intra-rater reliability was found for both raters across all measures. Most quantitative measures demonstrated excellent or almost excellent reliability, with the exception of the thumb width item for rater 1 and the last shape item for rater 2. Intra-rater kappa and percentage agreement statistics for categorical measures from the tool are shown in Table 3. With three exceptions, all items were found to possess at least moderate intra-rater reliability for both raters. However, the three that did not (adequate depth, upper wear pattern, and outsole wear pattern) all demonstrated high percentage agreements (92 to 98%). This indicates the presence of the high agreement-low kappa paradox which can result if a low prevalence of some scores exists [51]. In these cases, the percentage agreement statistic provides a better indicator of overall agreement than the kappa statistic [51].

**Table 2:** Intra-rater reliability for continuous measures.

	Mean difference (95% LOA)		ICC	
	
Variable	Rater 1	Rater 2	Rater 1	Rater 2
*Fit*				
Foot length (mm)	0.3 (-2.5, 3.1)	0.3 (-3.9, 4.5)	0.99 (0.99 – 1.00)	0.99 (0.99 – 9.99)
Inside shoe length (straw) (mm)	0.5 (-7.7, 8.7)	-0.3 (-6.9, 6.3)	0.97 (0.94 – 0.99)	0.98 (0.96 – 0.99)
Inner shoe length – foot length (mm)	0.2 (-9.2, 9.6)	-0.6 (-9.2, 8.0)	0.78 (0.59 – 0.89)	0.81 (0.65 – 0.91)
Thumb width (mm)	-0.5 (-3.1, 2.1)	0.1 (-1.7, 1.9)	0.64 (0.24 – 0.86)	0.83 (0.58 – 0.94)
*General*				
Weight (g)	0.7 (-3.5, 4.9)	0.9 (-3.0, 4.8)	1.00 (1.00 – 1.00)	1.00 (1.00 – 1.00)
Length (mm)	0.9 (-5.2, 7.0)	1.7 (-6.8, 10.2)	0.99 (0.97 – 0.99)	0.97 (0.94 – 0.99)
Weight/length	0.00 (-0.02, 0.02)	0 (-0.02, 0.02)	1.00 (1.00 – 1.00)	1.00 (1.00 – 1.00)
*General structure*				
Heel height (mm)	-1 (-6, 4)	1 (-5, 7)	0.99 (0.97 – 0.99)	0.97 (0.94 – 0.99)
Forefoot height (mm)	0 (-4, 4)	0 (-3, 3)	0.95 (0.90 – 0.98)	0.97 (0.93 – 0.98)
Longitudinal profile (mm)	-1 (-5, 3)	1 (-5, 7)	0.99 (0.96 – 0.99)	0.97 (0.95 – 0.99)
Normaliaed longitudinal profile	-0.004 (-0.020, 0.012)	0.004 (-0.018, 0.026)	0.99 (0.97 – 1.00)	0.98 (0.96 – 0.99)
Last shape	-0.2 (-3.0, 2.6)	0.2 (-4.0, 4.4)	0.86 (0.72 – 0.93)	0.65 (0.39 – 0.82)
*Motion control properties*				
Number of laces	-0.1 (-0.6, 0.4)	-0.1 (-0.6, 0.4)	0.96 (0.90 – 0.99)	0.97 (0.91 – 0.99)
Motion control scale	0.1 (-1.8, 2.0)	0.3 (-1.9, 2.5)	0.93 (0.86 – 0.97)	0.91 (0.83 – 0.96)
*Cushioning*				
Midsole durometer	-1.2 (-9.2, 6.8)	0.1 (-9.9, 10.1)	0.97 (0.94 – 0.99)	0.95 (0.90 – 0.98)
Heel sole durometer	0.3 (-8.8, 9.4)	0.0 (-14.3, 14.3)	0.97 (0.93 – 0.98)	0.93 (0.85 – 0.96)

**Table 3:** Intra-rater reliability for categorical measures.

	% agreement		Kappa	
	
Variable	Rater 1	Rater 2	Rater 1	Rater 2
*Fit*				
Adequate length (palpation)	93	83	0.86	0.41
Adequate length (straw method)	83	77	0.65	0.51
Adequate width	92	93	0.65	0.69
Adequate depth	97	93	0.78	0.00*
*General*				
Age of shoe	97	97	0.92	0.92
Type of shoe	100	100	1.00	1.00
Upper materials	100	98	1.00	0.98
Outsole materials	97	100	0.65	1.00
*General structure*				
Heel height	98	95	0.94	0.83
Forefoot height	93	97	0.78	0.87
Longitudinal profile	100	88	1.00	0.66
Last shape	97	97	0.48	0.65
Fixation (outersole to midsole)	87	85	0.73	0.70
Sole flexion point	98	98	0.91	0.92
*Motion control properties*				
Dual density presence	100	100	1.00	1.00
Fixation (upper to foot)	100	98	1.00	0.93
Heel counter stiffness	96	96	0.87	0.86
Midfoot sagittal stability	92	88	0.78	0.71
Midfoot torsional stability	90	91	0.72	0.67
*Cushioning*				
Cushioning system presence	98	98	0.94	0.95
Lateral/medial midsole hardness	97	98	0.91	0.94
Heel-foot contact hardness	98	95	0.96	0.85
*Wear patterns*				
Upper wear pattern	98	92	0.85	-0.03*
Midsole wear pattern	100	100	1.00	1.00
Tread pattern	100	100	1.00	1.00
Tread wear	100	100	1.00	1.00
Outsole wear pattern	93	92	0.71	0.22*

* high-agreement -low kappa paradox

### Inter-rater reliability

Inter-rater ICCs and 95% LOAs for quantitative measures are shown in Table 4. The ICC results indicated generally similar reliability between therapists for both days. Excellent reliability was indicated on both days for all measures with the exception of the thumb width and last shape items which demonstrated poor to fair reliability on both days. Inter-rater kappa and percentage agreement statistics for categorical measures from the tool are shown in Table 5. With the exception of three items, all items were found to possess at least moderate inter-rater reliability for both testing sessions. Again these three items (adequate depth, upper wear pattern, and outsole wear pattern) demonstrated high percentage agreements (92 to 97%), indicating the presence of the high agreement-low kappa paradox [51].

**Table 4:** Inter-rater reliability for continuous measures.

	Mean difference (95% LOA)		ICC	
	
	Day 1	Day 2	Day 1	Day 2
*Fit*				
Foot length (mm)	1.2 (-1.1, 3.5)	1.1 (-0.8, 3.0)	0.99 (0.92 – 1.00)	0.99 (0.92 – 1.00)
Inside shoe length (straw) (mm)	0.1 (-7.5, 7.7)	-0.7 (-6.6, 5.2)	0.98 (0.95 – 0.99)	0.99 (0.97 – 0.99)
Inner shoe length – foot length (mm)	-1.1 (-9.1, 6.9)	-1.9 (-8.6, 4.8)	0.83 (0.67 – 0.91)	0.87 (0.67 – 0.94)
Thumb width (mm)	-0.2 (-2.7, 2.3)	0.5 (-1.9, 2.9)	0.69 (0.30 – 0.89)	0.69 (0.31 – 0.88)
*General*				
Weight (g)	-0.3 (-2.9, 2.3)	-0.1 (-3.7,3.5)	1.00 (1.00 – 1.00)	1.00 (1.00 – 1.00)
Length (mm)	-0.8 (-7.0, 5.4)	0 (8.6)	0.99 (0.97 – 0.99)	0.97 (0.95 – 0.99)
Weight/length	0.00 (-0.01, 0.01)	0.00 (-0.02, 0.02)	1.00 (1.00 – 1.00)	1.00 (1.00 – 1.00)
*General structure*				
Heel height (mm)	1 (-7, 9)	4 (-5, 13)	0.96 (0.91 – 0.98)	0.97 (0.95 – 0.99)
Forefoot height (mm)	0 (-4, 4)	1 (-4, 6)	0.94 (0.87 – 0.97)	0.90 (0.80 – 0.95)
Longitudinal profile (mm)	1 (-5, 7)	4 (-7,15)	0.97 (0.94 – 0.99)	0.91 (0.74 – 0.96)
Normalised longitudinal profile	0.005 (-0.019, 0.029)	0.013 (-0.028, 0.054)	0.98 (0.95 – 0.99)	0.92 (0.78 – 0.97)
Last shape	0.8 (-2.9, 4.5)	1.2 (-2.5, 4.9)	0.74 (0.49 – 0.87)	0.63 (0.25 – 0.82)
*Motion control properties*				
Number of laces	-0.1 (-0.6, 0.4)	-0.1 (-0.6, 0.4)	0.96 (0.90 – 0.99)	0.97 (0.91 – 0.99)
Motion control scale	0.0 (-2.0, 2.0)	0.2 (-1.5, 1.9)	0.93 (0.85 – 0.96)	0.95 (0.89 – 0.98)
*Cushioning*				
Midsole durometer	-1.7 (-11.9, 8.5)	-0.4 (-7.1, 6.3)	0.95 (0.90 – 0.98)	0.98 (0.96 – 0.99)
Heel sole durometer	0.4 (-12.5, 13.3)	0.1 (-14.6, 14.8)	0.93 (0.86 – 0.97)	0.92 (0.83 – 0.96)

**Table 5:** Inter-rater reliability for categorical measures.

	% agreement		Kappa	
	
Variable	Day 1	Day 2	Day 1	Day 2
*Fit*				
Adequate length (palpation)	88	83	0.59	0.66
Adequate length (straw method)	97	90	0.93	0.79
Adequate width	92	97	0.68	0.82
Adequate depth	97	93	0.78	0.00*
*General*				
Age of shoe	100	100	1.00	1.00
Type of shoe	98	98	0.93	0.93
Upper materials	95	93	0.87	0.82
Outsole materials	100	97	1.00	0.65
*General structure*				
Heel height	95	92	0.83	0.71
Forefoot height	93	93	0.76	0.77
Longitudinal profile	95	83	0.81	0.51
Last shape	98	100	0.79	1.00
Fixation (Outersole to midsole)	85	90	0.70	0.80
Sole flexion point	97	97	0.82	0.83
*Motion control properties*				
Dual density presence	100	100	1.00	1.00
Fixation (upper to foot)	85	90	1.00	0.93
Heel counter stiffness	93	96	0.81	0.86
Midfoot sagittal stability	88	95	0.69	0.87
Midfoot torsional stability	89	93	0.84	0.83
*Cushioning*				
Cushioning system presence	95	95	0.84	0.83
Lateral/medial midsole hardness	92	94	0.76	0.80
Heel-foot contact hardness	95	95	0.87	0.86
*Wear patterns*				
Upper wear pattern	97	93	0.73	-0.03*
Midsole wear pattern	100	100	1.00	1.00
Tread pattern	100	100	1.00	1.00
Tread wear	100	100	1.00	1.00
Outsole wear pattern	93	92	0.63	0.38*

* high-agreement -low kappa paradox

## Discussion

Footwear characteristics are considered important for treatment and prevention in various patient populations. However, previously there have been very few objective tools available for use clinically or for research purposes. Tools which have been previously published [17-20] have lacked evaluation of their reliability, or their applicability has been limited to a specific population. In this study, the *Footwear Assessment Tool *was developed as a comprehensive tool potentially applicable to a range of populations in clinical and research settings. The tool when completed in its entirety takes around 10 minutes, although this time is shortened with experience or by omitting components the researcher or therapist may believe are irrelevant to their patient(s). Each item within the tool was evaluated for reliability, and this along side consideration of validity for further use clinically and in research will now be discussed.

### Item 1. Fit

Inadequately sized shoes have been reported in between 72 and 81% of older people [23,25,27], 88% of females aged 20 to 60 years of age [24], and 80% of patients attending a general diabetic clinic [53]. However, the reliability of previous methods of assessing fit have not been reported. The current tool assessed three components of fit including length, width, and depth. Length was measured by both subjective palpation and more objectively using foot length and inner-shoe length measured with a flexible plastic straw. Both methods demonstrated at least moderate intra-rater and inter-rater reliability, indicating they can be applied in future research with some confidence.

When comparing reliability of the palpation and straw methods, rater 1 showed superior intra-rater reliability using the palpation method and rater 2 showed superior intra-rater reliability using the straw method. The straw method showed superior inter-rater reliability on both occasions. Although this indicates superior overall reliability for the straw method, clinical application and validity of the two measures needs to be considered. The palpation method is more efficient, and accounts for potential foot length changes due to altered foot posture caused by the shoe. The straw method is more time consuming, requires equipment, and does not account for any change to foot length as a result of altered foot posture. Interestingly, the palpation method revealed 14 out of 30 shoes to be too short compared to 19 out of 30 when using the straw method. This discrepancy may have resulted from changes to foot posture (e.g. more supinated) and overall length when the foot is placed in the shoe. Substantial reliability was found for intra-rater and inter-rater comparisons for both width and depth measurements from the current tool, with the exception of inter-rater reliability for depth measurement on day 2. However, percentage agreement was high (93%), indicating that all categorical items related to fit can be used in future research with confidence.

The LOA measurement errors for intra-rater (see Table 2) and inter-rater (see Table 4) comparisons were relatively large for the quantifiable measure of difference between foot length and inner shoe length (measured by the straw) when considered in context of the range of scores (-8 – 14 mm). This would indicate that future research to establish optimal footwear to foot length relationships in injury prevention and treatment may need to develop a more reliable method of measurement to that used in this study. Until further research is conducted on the clinical validity of both methods, the 'palpation' method is recommended due to its comparative reliability combined with superior efficiency.

### Item 2. General features

Almost all intra-rater and inter-rater comparisons for categorical items in the general section of the current tool demonstrated excellent reliability. The exceptions were the materials (outsole) item intra-rater reliability for rater 1 and inter- rater reliability on day 2. However, both of these comparisons showed high percentage agreement scores (97%). Weight, length, and weight/length ratio items all demonstrated excellent intra-rater and inter-rater reliability, and very low measurement errors compared to their respective ranges.

The current study indicated superior intra-rater (1.00 versus 0.70 to 1.00 [20]) and inter-rater (0.93 versus 0.80 to 0.90 [20]) reliability to that of Menz and Sherrington [20] for categorising footwear type. Improved reliability in the current study may have resulted from evaluating a larger range of footwear types and/or use of the picture chart (see Additional material file) to assist decision making. Therefore, the use of this chart in future research using this item is recommended.

### Item 3. General structure

Categorical measurements of heel height, forefoot height, and longitudinal profile demonstrated at least substantial intra-rater reliability and at least moderate inter-rater reliability for all measures. The percentage agreement statistic for categorizing heel height in the current study was similar to that reported by Menz and Sherrington [20]. Categories used in the current scale were based on consensus between researchers involved in this investigation and a previously published tool [20]. However the categories have not been validated, with the exception of one study indicating heel elevation greater than 2.5 cm was associated with hallux valgus and plantar calluses in older women [27]. Therefore, it is recommended quantitative measurements are recorded and used in future research to assist development of validated categories for specific populations. The most valid measurement to develop categories would be the normalised longitudinal profile (pitch) measure. This measure accounts for all properties which may affect the posture of the foot in the shoe. Excellent intra-rater and inter-rater reliability was demonstrated for quantitative measurements of heel height, forefoot height, and longitudinal profile. Measurement errors compared to the range for each quantitative measure were also very low.

All three remaining items from the general structure component of the tool (last shape, fixation of upper to sole, and forefoot sole flexion point) demonstrated at least substantial intra-rater and inter-rater reliability, with the exception of intra-rater reliability of rater 1 for last shape. However, this comparison showed a high percentage agreement statistic (97%). When reliability for sole flexion point in the current study is compared to that reported by Menz and Sherrington [20] for the same item, similar inter-rater reliability (0.82 to 0.83 versus 0.75 to 1.00 [20]), and superior intra-rater reliability (0.91 to 0.92 versus 0.40 to 0.62 [20]). Superior intra-rater reliability may have been the result of larger number of shoes and subsequently a greater number of scores for each category.

Intra-class correlation coefficient (ICC) results indicate that using a more quantifiable measure of last shape (performed after visually categorising the last) possesses only poor to good intra-rater (0.65 to 0.86) and poor to moderate inter-rater (0.63 to 0.74) reliability. The LOA measurement errors for intra-rater (see Table 2) and inter-rater (see Table 4) were also high compared to the range of the measure (0 – 14°). Therefore, it is recommended this quantitative measurement technique be used with caution in future research.

### Item 4. Motion control properties

All motion control properties categorical items in the current study demonstrated at least substantial reliability for both intra-rater and inter-rater comparisons. The *Footwear Assessment Form *[20] contained three similar motion control property items. These included fixation (fixation of the upper to the foot), heel counter stiffness, and longitudinal sole rigidity (midfoot sagittal stability). Kappa statistics were used to compare fixation and heel counter stiffness reliability reported in Menz and Sherrington's [20] results. However, midfoot sagittal stability (longitudinal sole rigidity) was compared using percentage agreement statistics due to a high agreement – low kappa paradox in Menz and Sherrington's [20] results. Midfoot sagittal stability results in the current study indicated inferior intra-rater reliability (88 to 92% versus 92 to 100% [20]), and similar inter-rater reliability (88 to 95% versus 92% [20]). Fixation (upper to foot) in the current study showed similar intra-rater reliability (0.93 to 1.00 versus 0.73 to 1.00 [20]) and superior inter-rater reliability (0.93 to 1.00 versus 0.87 [20]). Heel counter stiffness showed superior intra-rater (0.86 to 0.87 versus 0.77 to 0.86 [20]) and inter-rater (0.81 to 0.86 versus 0.64 to 0.75 [20]) reliability.

Slightly better overall reliability in the present study may have resulted from testing a larger number of shoes (30 versus 12 [20]) and a subsequent improvement in reliability with experience. Although differences existed between this study and that by Menz and Sherrington [20], reliability in both studies for each item was high, strengthening the claim of these items to possess adequate reliability for future use.

### Motion control properties scale

There is currently limited evidence that improving motion control properties of footwear can treat or prevent musculoskeletal injury [54]. The effect of motion control properties on clinical outcomes with foot orthoses is also limited. In order to thoroughly investigate these possible relationships, a reliable tool to assess overall motion control quality has been developed. The scale demonstrated excellent intra-rater and inter-rater reliability, and reasonable measurement error scores between days (see Table 2) and between raters (see Table 4) when compared to the range of possible scores (0 to 11). These findings, combined with high reliability for each individual item, would indicate the scale can be used both clinically and in future research with confidence. However, despite good face validity, the scale and each item lack good quality research to support their clinical validity. Firstly, current motion control property items included in the scale are based on general consensus within the literature. Secondly, categories for subjective measures of heel counter stiffness, midfoot sole sagittal stability and midfoot sole torsional stability items are based on arbitrary ranges (i.e. 0 – 10°, 10 – 45°, and > 45°). Therefore, further research is needed to evaluate injury risk and treatment of various patient populations with footwear containing various characteristics from within the scale. This will allow the clinical validity of each item and the scale to be evaluated and modified if appropriate.

### Item 5. Cushioning

Optimal footwear characteristics related to cushioning to treat and prevent musculoskeletal injury or prevent falls are currently unclear [54]. To evaluate possible relationships, reliable techniques to assess characteristics are needed. Excellent intra-rater and inter-rater reliability was found for all categorical cushioning items with the exception of inter-rater reliability for lateral/medial midsole hardness on day 1 which showed substantial reliability. Although high reliability for these items is indicated, the clinical validity of the categories within each item still needs to be evaluated on various patient populations.

Findings from the current study indicate much better reliability compared to findings reported by Menz and Sherrington [20] for heel sole hardness. Menz and Sherrington [20] reported moderate to substantial intra-rater reliability and poor to moderate inter-rater reliability, compared to excellent intra-rater and inter-rater reliability in the current study. Superior reliability in the current study may have resulted from more detailed descriptions of each category (i.e. soft, firm, or hard).

Menz and Sherrington [20] recommended using a more objective measure such as the Shore A standard test for durometer hardness to measure material density. Reliability of this method in the current study indicated excellent intra-rater and inter-rater reliability for midsole and heel sole hardness. Heel sole hardness durometer measurement showed reasonable LOA measurement errors for intra-rater (see Table 2) and inter-rater (see Table 4) comparisons when compared to the range (10 – 87). Midsole hardness durometer measurement also showed reasonable measurement errors for intra-rater (see Table 2) and inter-rater (see Table 4) comparisons when compared to the range (34 – 100). Considering these strong reliability findings, objective Shore A durometer measurements of footwear material density may be a more valid measurement technique compared to subjective categorisation where quantification of density measurements is required. Further research is now needed to develop and validate quantifiable categories of material density for various patient populations. Until this is achieved, clinicians and researchers that do not have access to a penetrometer may use subjective evaluation of cushioning properties with confidence that it is a reliable alternative.

### Item 6. Wear patterns

All items from this section demonstrated substantial to significant intra-rater and inter-rater reliability with the exception of upper wear pattern and outsole wear pattern for intra-rater reliability for rater 2 and inter-rater reliability on day 2. However, both of these items showed high percentage agreements for intra-rater (92%) comparisons from rater 2 and inter-rater (92 – 93%) comparisons on day 2, indicating high agreement – low kappa paradoxes. These paradoxes resulted from the very low number of scores from both raters outside of normal for these items. Since this study was conducted on a non-clinical population, this low number of abnormal wear patterns is not surprising. Therefore, despite the apparent high reliability of upper, midsole, and outersole wear patterns, it is recommended these items are used with some caution until reliability can be established on footwear from clinical populations who are likely to produce abnormal wear patterns. Unfortunately, the lack of abnormal wear patterns prevented the addition of pictorial guidance for future research and clinical use of the tool. This is an addition to the tool that is recommended if it is applied in future research investigating the relationship between abnormal wear patterns and pathology.

## Conclusion

Optimal footwear characteristics in a range of patient populations remain unclear. The *Footwear Assessment Tool *was devised in an attempt to produce a comprehensive footwear assessment tool which is valid, reliable, and can be efficiently applied in clinical and research settings. Based on face validity and findings of high reliability for all categorical items, use of these items from the tool to assist clinical footwear assessment can be recommended for a range of populations. Qualitative evaluation of the tool and each of its components during its application by a range of clinicians in different patient populations may provide guidance for future improvements to the tool. Further research using more quantitative measures from the tool is also needed to assist evaluation of the clinical significance of categories from each item.

High reliability was found for all quantitative measures from the current tool with the exception of last shape, thumb width measurement, and the difference between shoe length and foot length. With the exception of these three items, use of the quantitative measures from the scale in future research aiming to optimise the clinical significance of categories within each item is justified. Achievement of this will require application of the tool to footwear of participants during clinical prediction rule studies aimed at establishing etiological factors of various conditions and possible factors related to successful treatment outcomes. Such research may allow development of a safe normalised longitudinal profile (heel height) and midsole material density for those at risk of falling, optimal material densities to prevent overuse injuries, and optimal foot length/shoe length relationship to prevent pressure areas in diabetic patients. The motion control properties scale has good face validity and high reliability. However, the clinical significance of each items inclusion and the weightings (score) for each category still requires evaluation.

## Competing interests

HBM is Editor-in-Chief of the *Journal of Foot and Ankle Research*. It is journal policy that editors are removed from the peer review and editorial decision making processes for papers they have coauthored.

## Authors' contributions

CJB coordinated data collection and analysis, and with DB collected all data. All authors were involved in the development of the scale and the interpretation of the results, helped draft the manuscript, and read and approved the final manuscript.

## Supplementary Material

Additional file 1 **Development and evaluation of a tool for the assessment of footwear characteristics compressed folder**. The compressed folder contains a web links to the footwear assessment tool, the motion control scale, pictures related to each assessment item from the tool, and pictures to assist categorization of footwear type. Click here for file
