# Supplementary material for: Development and evaluation of a tool for the assessment of footwear characteristics
Source: J Foot Ankle Res. 2009 Apr 23;2:10. doi: 10.1186/1757-1146-2-10 (PMC2678108; doi:10.1186/1757-1146-2-10)
Supplement: Additional file 1 — Development and evaluation of a tool for the assessment of footwear characteristics compressed folder. The compressed folder contains a web links to the footwear assessment tool, the motion control scale, pictures related to each assessment item from the tool, and pictures to assist categorization of footwear type. [file 1757-1146-2-10-S1.zip › Additional_material/All_figures_combined.pdf]

Development and evaluation of a tool for the assessment of footwear characteristics

Christian J. Barton, Daniel Bonanno, Hylton B. Menz

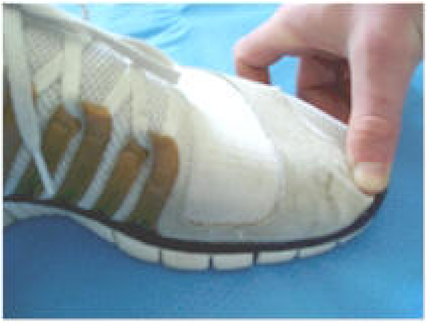

Palpation of footwear length

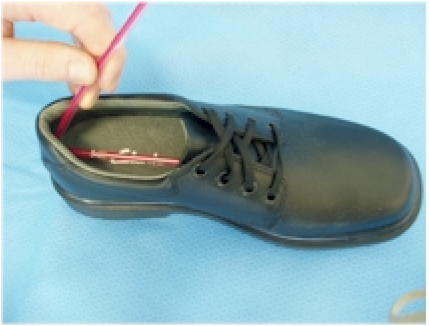

Straw method of measuring footwear length (A)

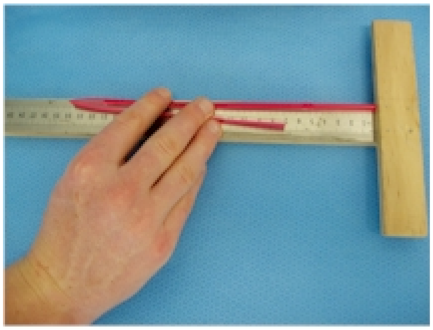

Straw method of measuring footwear length (B)

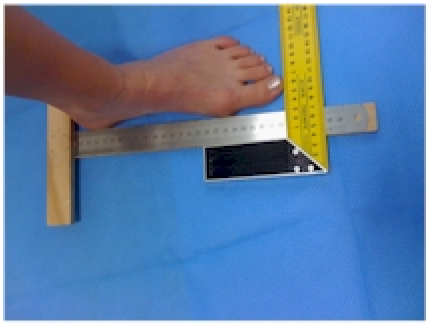

Custom-built Brannock-style device

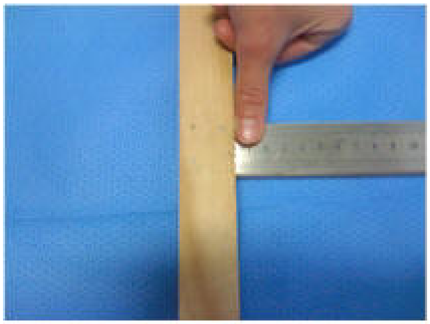

Measurement of thumb width

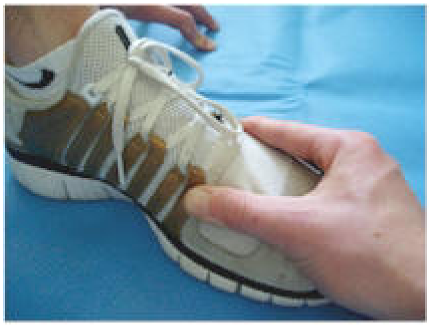

Measurement of footwear width

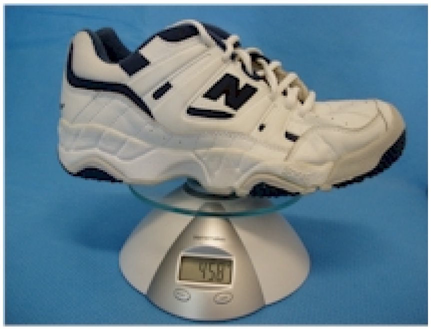

Measurement of footwear weight

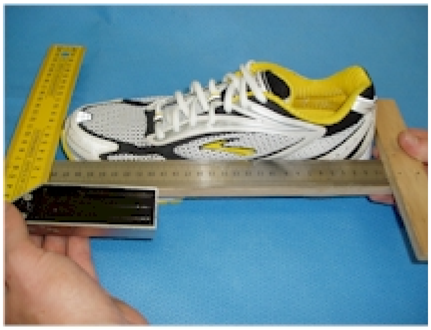

Measurement of footwear length using custom built Brannock-style device

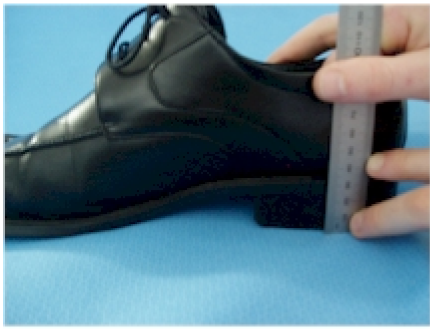

Measurement of heel height

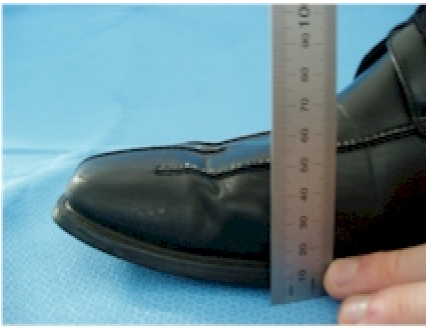

Measurement of forefoot height

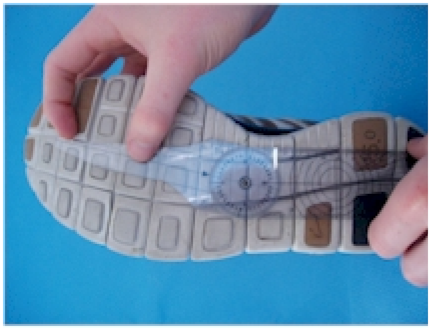

Measurement of last shape

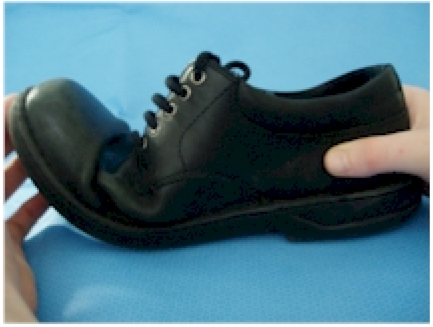

Measurement of sole flexion point

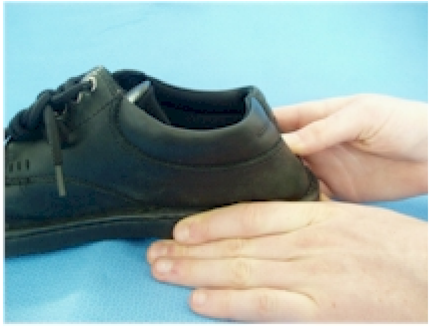

Measurement of heel counter stiffness

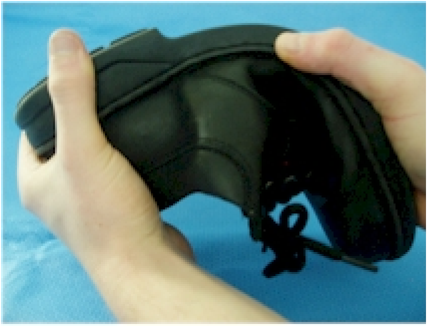

Measurement of midfoot sole sagittal stability

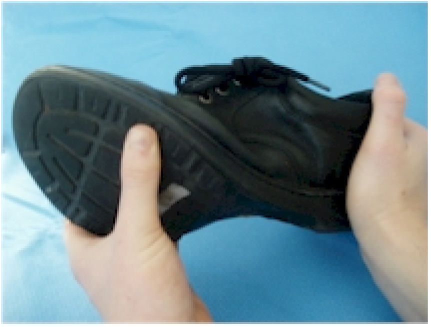

Measurement of midfoot sole torsional stability

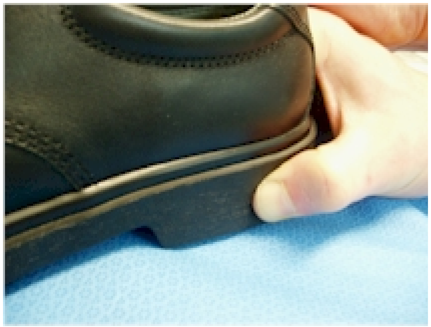

Subjective measurement of lateral midsole hardness

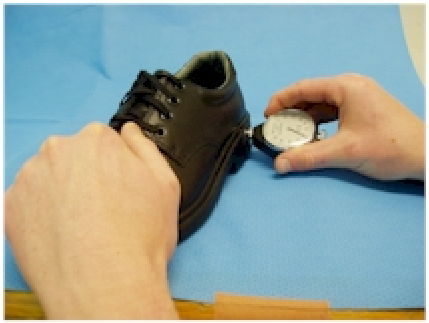

Measurement of lateral midsole hardness using a penetrometer

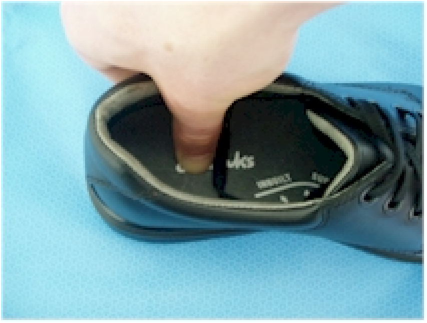

Subjective measurement of heel sole hardness
