# Supplementary material for: Development and evaluation of a tool for the assessment of footwear characteristics
Source: J Foot Ankle Res. 2009 Apr 23;2:10. doi: 10.1186/1757-1146-2-10 (PMC2678108; doi:10.1186/1757-1146-2-10)
Supplement: Additional file 1 — Development and evaluation of a tool for the assessment of footwear characteristics compressed folder. The compressed folder contains a web links to the footwear assessment tool, the motion control scale, pictures related to each assessment item from the tool, and pictures to assist categorization of footwear type. [file 1757-1146-2-10-S1.zip › Additional_material/Footwear_assessment_tool.pdf]

Appendix 1: FOOTWEAR ASSESSMENT TOOL

1. FIT

Foot length ☐ ***Thumb width*** ☐

Fit of shoe (length) – rule of thumb (wearer's thumb)

Palpation:	good ☐	too short (< ½ thumb) ☐	too long (> 1 ½) ☐
Straw =	good ☐	too short (< ½ thumb) ☐	too long (> 1 ½) ☐

Fit of shoe (width) – grasp test	good ☐	too narrow ☐	too wide ☐
Fit of shoe (depth)	good ☐	too shallow ☐	

2. GENERAL

Age of shoe	0 – 6 months ☐	6 – 12 months ☐	> 12 months ☐

Footwear style

walking shoe ☐	athletic shoe ☐	oxford shoe ☐	moccasin ☐
boot ☐	ugg-boot ☐	high heel ☐	Thong/flip-flop ☐
slipper ☐	backless slipper ☐	court shoe ☐	mule ☐
sandal ☐	surgical/bespoke ☐	other (specify) ☐	

Materials (upper)	leather ☐	synthetic ☐	mesh ☐	other ☐
Materials (outsole	rubber ☐	plastic ☐	leather ☐	other ☐

Weight ☐ ***Length*** ☐ ***Weight/length*** ☐

3. GENERAL STRUCTURE

Heel height =

0 – 2.5 cm ☐	2.6 – 5.0 cm ☐	> 5.0 cm ☐

Forefoot height (measured at point of the 1st and MTPJs) =

0 – 0.9 cm ☐	1.0 – 2.0 cm ☐	> 2.0 cm ☐

Longitudinal profile (heel – forefoot difference) =

flat (0 – 0.9 cm) ☐	small heel rise (1 – 3 cm) ☐	large heel rise (> 3 cm) ☐

Last (centre goniometer at 50% shoe length) =

straight (< 5°) ☐	semi-curved (5 – 15°) ☐	curved (> 15°) ☐

Fixation of upper to sole

board ☐	combination ☐	slip-lasted ☐

Forefoot sole flexion point

at level of MTPJs ☐	proximal to 1st MTPJ ☐	distal to 1st MTPJ ☐

4. MOTION CONTROL PROPERTIES

Density single ☐ dual ☐

Fixation none ☐ laces ☐ Number of eyelets ☐ straps/buckles ☐ Velcro ☐ zips ☐

Heel counter stiffness (20mm above bottom or upper)

no heel counter ☐ minimal (> 45°) ☐ moderate (< 45°) ☐ rigid (0-10°) ☐

Midfoot sole sagittal stability

minimal (> 45°) ☐ moderate (< 45°) ☐ rigid (0-10°) ☐

Midfoot sole frontal stability (torsional)

minimal (> 45°) ☐ moderate (< 45°) ☐ rigid (0-10°) ☐

5. CUSHIONING

Presence none ☐ heel ☐ heel/forefoot ☐

Lateral Midsole hardness	soft		firm		hard			
Durometer readings	1st		2nd		3rd		mean	
Medial Midsole hardness	soft		firm		hard			
Durometer readings	1st		2nd		3rd		mean	
Heel sole hardness (centre of inside heel shoe interface)	soft		firm		hard			
Durometer readings	1st		2nd		3rd		mean	

6. WEAR PATTERNS

Upper medial tilt (> 10°) ☐ neutral ☐ lateral tilt (> 10°) ☐

Midsole medial compression signs ☐ neutral ☐ lateral compression signs ☐

Tread pattern
A textured ☐ smooth (i.e. no pattern) ☐
B not worn ☐ partly worn ☐ fully worn ☐

Outsole wear pattern none ☐ normal ☐ lateral ☐ medial ☐

R

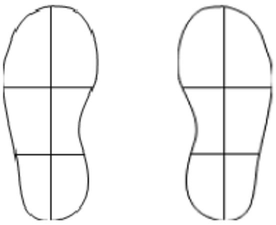

L
