# Supplementary material for: Development and evaluation of a tool for the assessment of footwear characteristics
Source: J Foot Ankle Res. 2009 Apr 23;2:10. doi: 10.1186/1757-1146-2-10 (PMC2678108; doi:10.1186/1757-1146-2-10)

Walking shoe

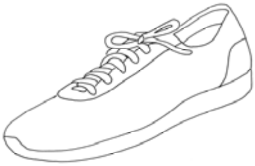

Athletic shoe / Runner

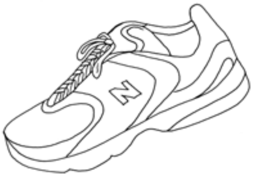

Oxford shoe

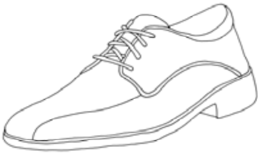

Moccassin

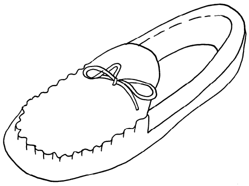

Boot

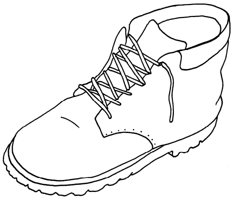

Ugg boot

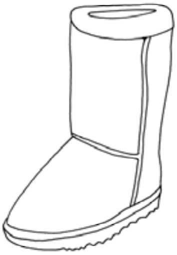

High heel / Stiletto

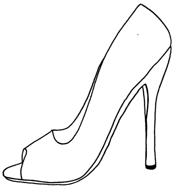

Thong / Flip flop

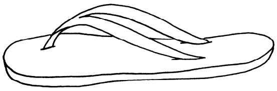

Slipper

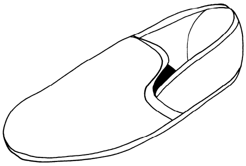

Backless slipper

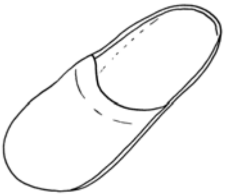

Court shoe

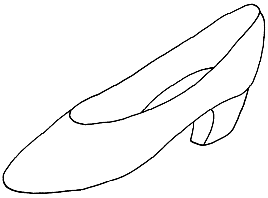

Mule

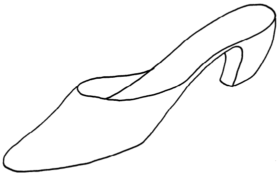

Sandal

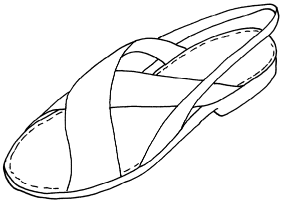

Surgical / Bespoke footwear

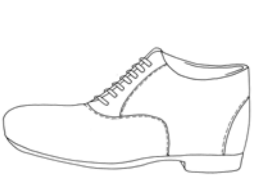

Supplement: Additional file 1 — Development and evaluation of a tool for the assessment of footwear characteristics compressed folder. The compressed folder contains a web links to the footwear assessment tool, the motion control scale, pictures related to each assessment item from the tool, and pictures to assist categorization of footwear type. [file 1757-1146-2-10-S1.zip › Additional_material/Footwear_type_picture_chart.pdf]
