# Supplementary material for: Development and evaluation of a tool for the assessment of footwear characteristics
Source: J Foot Ankle Res. 2009 Apr 23;2:10. doi: 10.1186/1757-1146-2-10 (PMC2678108; doi:10.1186/1757-1146-2-10)
Supplement: Additional file 1 — Development and evaluation of a tool for the assessment of footwear characteristics compressed folder. The compressed folder contains a web links to the footwear assessment tool, the motion control scale, pictures related to each assessment item from the tool, and pictures to assist categorization of footwear type. [file 1757-1146-2-10-S1.zip › Additional_material/index.html]

Footwear assessment tool


**Development and evaluation of a tool for the
assessment of footwear characteristics**

Christian J. Barton, Daniel Bonanno, Hylton B.
Menz

**Additional material**

|  |
| --- |
| Footwear Assessment Tool |
| Footwear type picture chart |
| Motion control properties scale |
| All figures combined |

**Figures demonstrating each assessment item**

|  |  |  |
| --- | --- | --- |
|  |  |  |
| Palpation of footwear length | Straw method of measuring footwear length (A) | Straw method of measuring footwear length (B) |
|  |  |  |
| Custom-built Brannock-style device | Measurement of thumb width | Measurement of footwear width |
|  |  |  |
| Measurement of footwear weight | Measurement of footwear length using custom built Brannock-style device | Measurement of heel height |
|  |  |  |
| Measurement of forefoot height | Measurement of last shape | Measurement of sole flexion point |
|  |  |  |
| Measurement of heel counter stiffness | Measurement of midfoot sole sagittal stability | Measurement of midfoot sole torsional stability |
|  |  |  |
| Subjective measurement of lateral midsole hardness | Measurement of lateral midsole hardness using a penetrometer | Subjective measurement of heel sole hardness |
