# Supplementary material for: Development and evaluation of a tool for the assessment of footwear characteristics
Source: J Foot Ankle Res. 2009 Apr 23;2:10. doi: 10.1186/1757-1146-2-10 (PMC2678108; doi:10.1186/1757-1146-2-10)
Supplement: Additional file 1 — Development and evaluation of a tool for the assessment of footwear characteristics compressed folder. The compressed folder contains a web links to the footwear assessment tool, the motion control scale, pictures related to each assessment item from the tool, and pictures to assist categorization of footwear type. [file 1757-1146-2-10-S1.zip › Additional_material/Motion_control_properties_scale.pdf]

MOTION CONTROL PROPERTIES SCALE

	Score			
Item	0	1	2	3
Midsole density layers	Single density		Dual density	
Fixation (upper to foot)	None	Alternative to laces (e.g. strap, Velcro, zip, etc.)	Laces (at least 3 eyelets)	
Heel counter stiffness	No heel counter	Minimal	Moderate	Rigid
Midfoot sagittal stability	Minimal	Moderate	Rigid	
Midfoot torsional stability	Minimal	Moderate	Rigid	
