# Supplementary figures and images for: Development and evaluation of a tool for the assessment of footwear characteristics
Source: J Foot Ankle Res. 2009 Apr 23;2:10. doi: 10.1186/1757-1146-2-10 (PMC2678108; doi:10.1186/1757-1146-2-10)

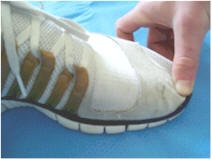

Supplement: Additional file 1 — Development and evaluation of a tool for the assessment of footwear characteristics compressed folder. The compressed folder contains a web links to the footwear assessment tool, the motion control scale, pictures related to each assessment item from the tool, and pictures to assist categorization of footwear type. [file 1757-1146-2-10-S1.zip › Additional_material/1.jpg]

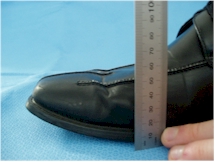

Supplement: Additional file 1 — Development and evaluation of a tool for the assessment of footwear characteristics compressed folder. The compressed folder contains a web links to the footwear assessment tool, the motion control scale, pictures related to each assessment item from the tool, and pictures to assist categorization of footwear type. [file 1757-1146-2-10-S1.zip › Additional_material/10.jpg]

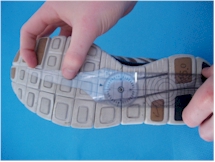

Supplement: Additional file 1 — Development and evaluation of a tool for the assessment of footwear characteristics compressed folder. The compressed folder contains a web links to the footwear assessment tool, the motion control scale, pictures related to each assessment item from the tool, and pictures to assist categorization of footwear type. [file 1757-1146-2-10-S1.zip › Additional_material/11.jpg]

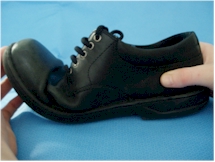

Supplement: Additional file 1 — Development and evaluation of a tool for the assessment of footwear characteristics compressed folder. The compressed folder contains a web links to the footwear assessment tool, the motion control scale, pictures related to each assessment item from the tool, and pictures to assist categorization of footwear type. [file 1757-1146-2-10-S1.zip › Additional_material/12.jpg]

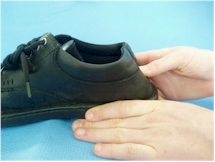

Supplement: Additional file 1 — Development and evaluation of a tool for the assessment of footwear characteristics compressed folder. The compressed folder contains a web links to the footwear assessment tool, the motion control scale, pictures related to each assessment item from the tool, and pictures to assist categorization of footwear type. [file 1757-1146-2-10-S1.zip › Additional_material/13.jpg]

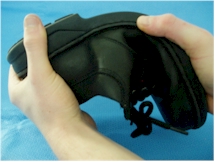

Supplement: Additional file 1 — Development and evaluation of a tool for the assessment of footwear characteristics compressed folder. The compressed folder contains a web links to the footwear assessment tool, the motion control scale, pictures related to each assessment item from the tool, and pictures to assist categorization of footwear type. [file 1757-1146-2-10-S1.zip › Additional_material/14.jpg]

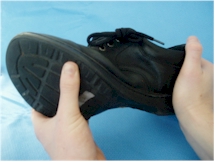

Supplement: Additional file 1 — Development and evaluation of a tool for the assessment of footwear characteristics compressed folder. The compressed folder contains a web links to the footwear assessment tool, the motion control scale, pictures related to each assessment item from the tool, and pictures to assist categorization of footwear type. [file 1757-1146-2-10-S1.zip › Additional_material/15.jpg]

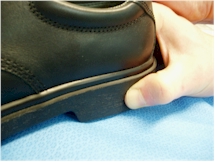

Supplement: Additional file 1 — Development and evaluation of a tool for the assessment of footwear characteristics compressed folder. The compressed folder contains a web links to the footwear assessment tool, the motion control scale, pictures related to each assessment item from the tool, and pictures to assist categorization of footwear type. [file 1757-1146-2-10-S1.zip › Additional_material/16.jpg]

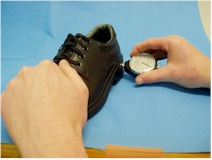

Supplement: Additional file 1 — Development and evaluation of a tool for the assessment of footwear characteristics compressed folder. The compressed folder contains a web links to the footwear assessment tool, the motion control scale, pictures related to each assessment item from the tool, and pictures to assist categorization of footwear type. [file 1757-1146-2-10-S1.zip › Additional_material/17.jpg]

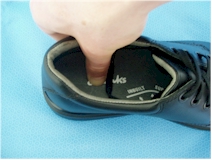

Supplement: Additional file 1 — Development and evaluation of a tool for the assessment of footwear characteristics compressed folder. The compressed folder contains a web links to the footwear assessment tool, the motion control scale, pictures related to each assessment item from the tool, and pictures to assist categorization of footwear type. [file 1757-1146-2-10-S1.zip › Additional_material/18.jpg]

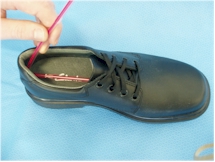

Supplement: Additional file 1 — Development and evaluation of a tool for the assessment of footwear characteristics compressed folder. The compressed folder contains a web links to the footwear assessment tool, the motion control scale, pictures related to each assessment item from the tool, and pictures to assist categorization of footwear type. [file 1757-1146-2-10-S1.zip › Additional_material/2.jpg]

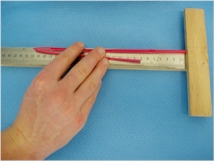

Supplement: Additional file 1 — Development and evaluation of a tool for the assessment of footwear characteristics compressed folder. The compressed folder contains a web links to the footwear assessment tool, the motion control scale, pictures related to each assessment item from the tool, and pictures to assist categorization of footwear type. [file 1757-1146-2-10-S1.zip › Additional_material/3.jpg]

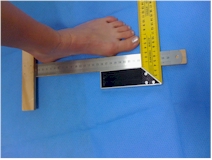

Supplement: Additional file 1 — Development and evaluation of a tool for the assessment of footwear characteristics compressed folder. The compressed folder contains a web links to the footwear assessment tool, the motion control scale, pictures related to each assessment item from the tool, and pictures to assist categorization of footwear type. [file 1757-1146-2-10-S1.zip › Additional_material/4.jpg]

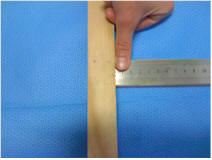

Supplement: Additional file 1 — Development and evaluation of a tool for the assessment of footwear characteristics compressed folder. The compressed folder contains a web links to the footwear assessment tool, the motion control scale, pictures related to each assessment item from the tool, and pictures to assist categorization of footwear type. [file 1757-1146-2-10-S1.zip › Additional_material/5.jpg]

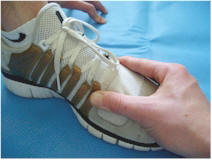

Supplement: Additional file 1 — Development and evaluation of a tool for the assessment of footwear characteristics compressed folder. The compressed folder contains a web links to the footwear assessment tool, the motion control scale, pictures related to each assessment item from the tool, and pictures to assist categorization of footwear type. [file 1757-1146-2-10-S1.zip › Additional_material/6.jpg]

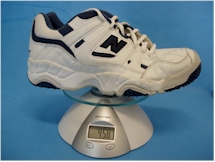

Supplement: Additional file 1 — Development and evaluation of a tool for the assessment of footwear characteristics compressed folder. The compressed folder contains a web links to the footwear assessment tool, the motion control scale, pictures related to each assessment item from the tool, and pictures to assist categorization of footwear type. [file 1757-1146-2-10-S1.zip › Additional_material/7.jpg]

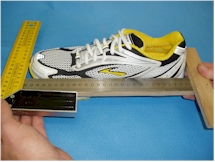

Supplement: Additional file 1 — Development and evaluation of a tool for the assessment of footwear characteristics compressed folder. The compressed folder contains a web links to the footwear assessment tool, the motion control scale, pictures related to each assessment item from the tool, and pictures to assist categorization of footwear type. [file 1757-1146-2-10-S1.zip › Additional_material/8.jpg]

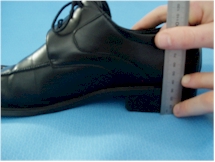

Supplement: Additional file 1 — Development and evaluation of a tool for the assessment of footwear characteristics compressed folder. The compressed folder contains a web links to the footwear assessment tool, the motion control scale, pictures related to each assessment item from the tool, and pictures to assist categorization of footwear type. [file 1757-1146-2-10-S1.zip › Additional_material/9.jpg]

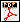

Supplement: Additional file 1 — Development and evaluation of a tool for the assessment of footwear characteristics compressed folder. The compressed folder contains a web links to the footwear assessment tool, the motion control scale, pictures related to each assessment item from the tool, and pictures to assist categorization of footwear type. [file 1757-1146-2-10-S1.zip › Additional_material/acrobatsmall[1].gif]
